# Supplementary material for: Socioeconomic disparities in attention deficit hyperactivity disorder (ADHD) in Sweden: An intersectional ecological niches analysis of individual heterogeneity and discriminatory accuracy (IEN-AIHDA)
Source: PLoS One. 2023 Nov 20;18(11):e0294741. doi: 10.1371/journal.pone.0294741 (PMC10659213; doi:10.1371/journal.pone.0294741)
Supplement: S1 Table — Ranked. (DOCX) [file pone.0294741.s001.docx]

| **Complete Table 3**. Number of individuals (N) and Prevalence or Absolute Risk (AR of Attention Deficit Hyperactivity Disorder (ADHD) in the 96 intercategorical strata as well as prevalence ration (PR). | | | |
| --- | --- | --- | --- |
| **Multicategorial strata** | **N** | **AR (99% CI)** | **PR (99% CI)** |
| Male 10-14 Middle Immigrant | 1959 | 6.18 (4.86-6.73) | 17.18 (12.44-23.73) |
| Male 15-19 High Immigrant | 1282 | 5.62 (4.09-6.27) | 15.62 (10.72-22.76) |
| Male 15-19 Middle Immigrant | 2397 | 5.59 (4.45-6.04) | 15.55 (11.35-21.3) |
| Male 10-14 High Immigrant | 1449 | 5.04 (3.67-5.59) | 14.01 (9.63-20.38) |
| Male 10-14 Low Swedish | 105259 | 4.22 (4.06-4.27) | 11.74 (9.37-14.72) |
| Male 15-19 Low Swedish | 154354 | 3.92 (3.80-3.96) | 10.91 (8.71-13.66) |
| Male 20-24 High Immigrant | 1318 | 3.26 (2.13-3.68) | 9.07 (5.78-14.25) |
| Female 15-19 High Immigrant | 1229 | 3.17 (2.03-3.60) | 8.83 (5.52-14.10) |
| Male 5-9 Middle Immigrant | 1002 | 2.79 (1.63-3.24) | 7.77 (4.55-13.27) |
| Male 10-14 Middle Swedish | 81227 | 2.77 (2.63-2.81) | 7.71 (6.13-9.70) |
| Female 15-19 Middle Immigrant | 2345 | 2.64 (1.86-2.89) | 7.35 (4.95-10.92) |
| Male 10-14 Low Immigrant | 7493 | 2.54 (2.09-2.66) | 7.05 (5.27-9.43) |
| Male 25-29 Low Swedish | 61715 | 2.50 (2.34-2.54) | 6.95 (5.51-8.76) |
| Female 20-24 High Immigrant | 1121 | 2.50 (1.46-2.88) | 6.95 (4.07-11.86) |
| Male 15-19 Middle Swedish | 94164 | 2.48 (2.35-2.51) | 6.89 (5.48-8.67) |
| Male 20-24 Middle Immigrant | 4522 | 2.48 (1.92-2.63) | 6.89 (4.95-9.58) |
| Male 30-39 Low Swedish | 66683 | 2.46 (2.31-2.49) | 6.84 (5.42-8.62) |
| Male 20-24 Low Swedish | 133027 | 2.45 (2.34-2.47) | 6.81 (5.43-8.55) |
| Male 5-9 High Immigrant | 1026 | 2.34 (1.30-2.72) | 6.51 (3.68-11.51) |
| Female 10-14 Middle Immigrant | 1993 | 2.26 (1.49-2.50) | 6.28 (4.03-9.79) |
| Male 15-19 Low Immigrant | 12919 | 1.98 (1.68-2.05) | 5.51 (4.19-7.25) |
| Female 15-19 Low Swedish | 147318 | 1.97 (1.88-1.99) | 5.49 (4.37-6.90) |
| Male 5-9 Low Immigrant | 2375 | 1.73 (1.11-1.90) | 4.80 (3.03-7.60) |
| Female 20-24 Low Swedish | 141012 | 1.57 (1.49-1.59) | 4.37 (3.48-5.50) |
| Female 30-39 Low Swedish | 97357 | 1.57 (1.47-1.59) | 4.36 (3.46-5.50) |
| Female 25-29 Low Swedish | 74915 | 1.56 (1.45-1.59) | 4.35 (3.44-5.50) |
| Male 5-9 Low Swedish | 89215 | 1.54 (1.43-1.56) | 4.28 (3.39-5.40) |
| Male 10-14 High Swedish | 38969 | 1.42 (1.27-1.45) | 3.95 (3.09-5.07) |
| Male 15-19 High Swedish | 37272 | 1.34 (1.19-1.36) | 3.72 (2.89-4.77) |
| Female 10-14 Low Swedish | 99781 | 1.30 (1.21-1.31) | 3.61 (2.85-4.56) |
| Female 20-24 Middle Immigrant | 3931 | 1.17 (0.77-1.27) | 3.25 (2.10-5.05) |
| Male 20-24 Low Immigrant | 20907 | 1.15 (0.97-1.19) | 3.21 (2.43-4.23) |
| Female 15-19 Middle Swedish | 88261 | 1.13 (1.04-1.14) | 3.14 (2.48-3.98) |
| Male 20-24 Middle Swedish | 107751 | 1.11 (1.03-1.12) | 3.09 (2.44-3.90) |
| Male 40-49 Low Swedish | 107586 | 1.10 (1.02-1.11) | 3.06 (2.42-3.86) |
| Female 10-14 High Immigrant | 1568 | 1.02 (0.48-1.19) | 2.84 (1.44-5.61) |
| Male 5-9 Middle Swedish | 94366 | 0.99 (0.91-1'00) | 2.75 (2.17-3.48) |
| Male 25-29 Low Immigrant | 20005 | 0.87 (0.71-0.90) | 2.43 (1.81-3.27) |
| Female 15-19 Low Immigrant | 12205 | 0.87 (0.67-0.91) | 2.42 (1.73-3.38) |
| Male 25-29 High Immigrant | 2494 | 0.84 (0.44-0.95) | 2.34 (1.28-4.29) |
| Female 40-49 Low Swedish | 163011 | 0.81 (0.75-0.82) | 2.25 (1.78-2.84) |
| Male 30-39 Middle Swedish | 174500 | 0.80 (0.75-0.81) | 2.22 (1.76-2.81) |
| Male 20-24 High Swedish | 31925 | 0.77 (0.65-0.79) | 2.13 (1.62-2.81) |
| Female 10-14 Middle Swedish | 76402 | 0.75 (0.67-0.76) | 2.08 (1.63-2.67) |
| Male 25-29 Middle Swedish | 110387 | 0.70 (0.64-0.71) | 1.94 (1.53-2.47) |
| Female 20-24 Low Immigrant | 19963 | 0.69 (0.55-0.71) | 1.91 (1.40-2.61) |
| Female 20-24 Middle Swedish | 90921 | 0.69 (0.62-0.69) | 1.91 (1.49-2.44) |
| Male 25-29 Middle Immigrant | 9776 | 0.65 (0.46-0.69) | 1.82 (1.23-2.69) |
| Female 15-19 High Swedish | 35617 | 0.65 (0.55-0.67) | 1.82 (1.38-2.41) |
| Female 5-9 Middle Immigrant | 1414 | 0.64 (0.21-0.78) | 1.77 (0.73-4.30) |
| Female 10-14 Low Immigrant | 7277 | 0.63 (0.42-0.67) | 1.76 (1.13-2.73) |
| Male 50-59 Low Swedish | 85974 | 0.56 (0.50-0.57) | 1.56 (1.21-2.01) |
| Male 5-9 High Swedish | 61146 | 0.56 (0.48-0.57) | 1.55 (1.19-2.02) |
| Male 40-49 Middle Swedish | 188017 | 0.56 (0.51-0.56) | 1.55 (1.22-1.96) |
| Male 30-39 Low Immigrant | 38349 | 0.53 (0.44-0.54) | 1.48 (1.11-1.97) |
| Female 20-24 High Swedish | 25108 | 0.53 (0.42-0.54) | 1.46 (1.07-2.01) |
| Female 5-9 Low Swedish | 84179 | 0.48 (0.42-0.48) | 1.32 (1.02-1.71) |
| Female 30-39 Middle Swedish | 200208 | 0.46 (0.43-0.47) | 1.29 (1.02-1.64) |
| Female 5-9 High Immigrant | 1549 | 0.45 (0.13-0.57) | 1.26 (0.46-3.41) |
| Female 25-29 Middle Immigrant | 9355 | 0.44 (0.28-0.47) | 1.22 (0.77-1.93) |
| Female 50-59 Low Swedish | 87541 | 0.44 (0.38-0.44) | 1.22 (0.94-1.58) |
| Female 25-29 Low Immigrant | 23041 | 0.43 (0.33-0.45) | 1.21 (0.86-1.70) |
| Female 25-29 Middle Swedish | 105572 | 0.42 (0.37-0.43) | 1.18 (0.92-1.52) |
| Male 30-39 Middle Immigrant | 24593 | 0.42 (0.32-0.44) | 1.18 (0.84-1.65) |
| Female 30-39 Low Immigrant | 49759 | 0.37 (0.30-0.38) | 1.03 (0.77-1.38) |
| Male 40-49 Middle Immigrant | 28004 | 0.37 (0.28-0.38) | 1.02 (0.73-1.43) |
| Female 30-39 Middle Immigrant | 26251 | 0.37 (0.28-0.38) | 1.02 (0.72-1.44) |
| Female 40-49 Middle Swedish | 192234 | 0.36 (0.33-0.37) | 1.01 (0.79-1.28) |
| **Female 10-14 High Swedish** | **37267** | **0.36 (0.28-0.37)** | **Ref** |
| Male 25-29 High Swedish | 53137 | 0.34 (0.28-0.35) | 0.95 (0.71-1.27) |
| Male 40-49 Low Immigrant | 48856 | 0.34 (0.27-0.35) | 0.94 (0.70-1.27) |
| Female 25-29 High Immigrant | 1832 | 0.33 (0.08-0.42) | 0.91 (0.31-2.67) |
| Male 30-39 High Immigrant | 14763 | 0.31 (0.21-0.33) | 0.87 (0.56-1.35) |
| Female 5-9 Middle Swedish | 89063 | 0.30 (0.25-0.30) | 0.82 (0.63-1.08) |
| Male 50-59 Middle Swedish | 162048 | 0.29 (0.26-0.29) | 0.81 (0.63-1.04) |
| Female 5-9 Low Immigrant | 2409 | 0.25 (0.06-0.31) | 0.69 (0.24-2.03) |
| Female 25-29 High Swedish | 30770 | 0.24 (0.17-0.25) | 0.67 (0.46-0.97) |
| Female 30-39 High Immigrant | 13307 | 0.23 (0.14-0.25) | 0.65 (0.39-1.08) |
| Female 40-49 Middle Immigrant | 26961 | 0.23 (0.16-0.24) | 0.64 (0.43-0.95) |
| Female 40-49 Low Immigrant | 59666 | 0.22 (0.17-0.23) | 0.62 (0.45-0.84) |
| Female 50-59 Middle Swedish | 164388 | 0.21 (0.18-0.21) | 0.57 (0.44-0.74) |
| Male 30-39 High Swedish | 240097 | 0.19 (0.17-0.19) | 0.54 (0.42-0.69) |
| Male 50-59 Middle Immigrant | 25123 | 0.19 (0.13-0.20) | 0.53 (0.34-0.82) |
| Male 50-59 Low Immigrant | 39615 | 0.18 (0.13-0.19) | 0.51 (0.35-0.75) |
| Male 40-49 High Immigrant | 19831 | 0.18 (0.11-0.19) | 0.50 (0.31-0.82) |
| Female 40-49 High Immigrant | 15998 | 0.18 (0.10-0.19) | 0.49 (0.29-0.83) |
| Male 40-49 High Swedish | 236364 | 0.16 (0.14-0.16) | 0.44 (0.34-0.57) |
| Female 5-9 High Swedish | 57851 | 0.13 (0.10-0.14) | 0.37 (0.26-0.54) |
| Female 40-49 High Swedish | 152524 | 0.13 (0.11-0.13) | 0.36 (0.27-0.48) |
| Female 50-59 Low Immigrant | 38058 | 0.13 (0.08-0.13) | 0.35 (0.23-0.54) |
| Female 30-39 High Swedish | 156217 | 0.12 (0.10-0.12) | 0.33 (0.25-0.45) |
| Female 50-59 Middle Immigrant | 29176 | 0.12 (0.07-0.12) | 0.32 (0.20-0.53) |
| Male 50-59 High Swedish | 235261 | 0.09 (0.07-0.09) | 0.24 (0.18-0.32) |
| Male 50-59 High Immigrant | 20962 | 0.09 (0.04-0.09) | 0.24 (0.13-0.46) |
| Female 50-59 High Swedish | 218100 | 0.05 (0.04-0.05) | 0.15 (0.11-0.21) |
| Female 50-59 High Immigrant | 25934 | 0.03 (0.01-0.04) | 0.09 (0.03-0.22) |
