# Supplementary material for: Socioeconomic disparities in attention deficit hyperactivity disorder (ADHD) in Sweden: An intersectional ecological niches analysis of individual heterogeneity and discriminatory accuracy (IEN-AIHDA)
Source: PLoS One. 2023 Nov 20;18(11):e0294741. doi: 10.1371/journal.pone.0294741 (PMC10659213; doi:10.1371/journal.pone.0294741)
Supplement: Attachment — Submitted filename: Response to Reviewers.docx [file pone.0294741.s005.docx]

| 'Response to Reviewers' (rebuttal letter)  PONE-D-23-22871 (deadline Oct 28 2023 11:59PM)  Socioeconomic disparities in attention deficit hyperactivity disorder (ADHD) in Sweden: An intersectional ecological niches analysis of individual heterogeneity and discriminatory accuracy (IEN-AIHDA)  Lakshit Jain, MD  Academic Editor  PLOS ONE  Dear Editor,  Thank you very much for this opportunity and for transmitting to us your comments and the comments of the referees. We have considered and responded to all the commentaries as explaining in detail below. | |
| --- | --- |
| **EDITOR & REFEREES** | **AUTHORS** |
| **Journal Requirements:** |  |
| When submitting your revision, we need you to address these additional requirements. |  |
| 1. Please ensure that your manuscript meets PLOS ONE's style requirements, including those for file naming. The PLOS ONE style templates can be found at  https://journals.plos.org/plosone/s/file?id=wjVg/PLOSOne_formatting_sample_main_body.pdf and  https://journals.plos.org/plosone/s/file?id=ba62/PLOSOne_formatting_sample_title_authors_affiliations.pdf. | We have met PLOS ONE's style requirements, including those for file naming. |
| 2. In your Data Availability statement, you have not specified where the minimal data set underlying the results described in your manuscript can be found. PLOS defines a study's minimal data set as the underlying data used to reach the conclusions drawn in the manuscript and any additional data required to replicate the reported study findings in their entirety. All PLOS journals require that the minimal data set be made fully available. For more information about our data policy, please see http://journals.plos.org/plosone/s/data-availability.  "Upon re-submitting your revised manuscript, please upload your study’s minimal underlying data set as either Supporting Information files or to a stable, public repository and include the relevant URLs, DOIs, or accession numbers within your revised cover letter. For a list of acceptable repositories, please see http://journals.plos.org/plosone/s/data-availability#loc-recommended-repositories. Any potentially identifying patient information must be fully anonymized.  Important: If there are ethical or legal restrictions to sharing your data publicly, please explain these restrictions in detail. Please see our guidelines for more information on what we consider unacceptable restrictions to publicly sharing data: http://journals.plos.org/plosone/s/data-availability#loc-unacceptable-data-access-restrictions. Note that it is not acceptable for the authors to be the sole named individuals responsible for ensuring data access.  We will update your Data Availability statement to reflect the information you provide in your cover letter. | We have now created a fully anonymized minimal dataset containing the variables used in our final analyses, as well as the additional data required to replicate the reported study findings in their entirety. These files are attached as Supporting Information files.  The complete dataset is only available by contacting the National Board of Health and Welfare and Statistics Sweden.  We also inform on this issue in the cover letter. |
| 3. Please include captions for your Supporting Information files at the end of your manuscript, and update any in-text citations to match accordingly. Please see our Supporting Information guidelines for more information: http://journals.plos.org/plosone/s/supporting-information | We have done so. |
|  |  |
| **5. Review Comments to the Author** |  |
| **Reviewer #1** |  |
| 1. The study includes whole Sweden population as a sample and that is the biggest strength of this study. | Yes, we agree |
| 2. The core question in current world still remains, what is the reason for increased diagnosis of ADHD in recent years ? Whether its really more prevalent now compared to previous decades or just were undiagnosed so far? Author has made it clear that aim of this study is different but it can't be separated form the core issue and so this study should have comment on this question too. | Thank you for your comments, this is a very important question: What is the reason for increased diagnosis of ADHD? Even if this is not the aim of our study, we mention certain factors that *may* have contributed to an increased prevalence: “the influence of the pharmaceutical industry, an increased biological focus within psychiatry, transitions from ICD to DSM and the role of the Internet [32]”. Of course, our results can’t really answer the question whether ADHD really is more prevalent or just was more undiagnosed before. If we are to speculate, we think it’s both. The cognitive demands are high in contemporary society, and the increasing pressure on our frontal lobes can make people develop impairment to a greater extent (in relation to notions of ‘normal’ functioning in a certain time and place). But also, as we note in the introduction “Psychiatric diagnoses are flexible objects that arise, evolve, and even disappear, in line with historical processes [27-28].” This means that the same people that were considered having neurosis in the 70s, or burnout in the 90s, are now considered to have an AD(H)D, conceptualized as an underlying entity causing impairment in everyday life.  This is a complex topic that requires a thorough discussion and should therefor be considered a separate paper. In our discussion, we have tried to focus on different ways to conceptualize the socioeconomic disparities that we found in the results section. |
| **Reviewer #2:** |  |
| Very important study on a topic that highly controversial on both sides of the Atlantic! It's a luxury to have access to such huge national database as it can inform a growing trend without having to worry about sampling biases. | Thank you very much for your positive opinion. We do agree with the referee |
|  |  |
| The background well written; however, I wonder if there's a way to make it somewhat simpler to follow. It does go into more details which could be presented even more effectively during the discussion part. | We have made the introduction more concise by being less repetitive and by moving some sentences from the introduction to the discussion part. |
|  |  |
| I would also comment on the fact that demographic data often look at difference from one perspective- the patient's perspective. It is equally important to consider the impact of the clinician who is making the diagnosis of ADHD. As a clinician practicing in both community and university setting, I come across many individuals with complaints of "ADHD" that have had the diagnosis made by primary care providers (or pediatricians), who might have "met the criteria" if seen by specialists. I don't highlight this point to discuss who is better placed to make this diagnosis, but only to review how clinician access (High/ low social economical status, immigrant/ native populations) could also be a major player behind varying prevalence. If that does seem to be the case, perhaps training could also help bridge this gap. If this information is not readily available, perhaps it could be highlighted as a limitation. | We have highlighted that the impact of the clinician can be a key factor behind varying prevalence. The reviewer’s idea is really interesting, and we are considering to include the clinician dimension in an IEN framework in future research. |
|  |  |
| Another factor to consider will be grouping "immigrant" population in one cohort. Immigrant population can vary highly from the standpoint of cultural differences in seeking mental health treatment, level of tolerance in managing these symptoms or to approach them with discipline. | Yes, “immigrant” is a highly heterogenous category. We have now addressed this in the discussion by briefly problematizing the grouping of immigrants as a single categorization. |
|  |  |
| The overriding principle of how the diagnosis of ADHD essentially describes symptoms and groups them to "create" a medical condition, is problematic in my opinion. Looking through the same lens, diagnosis of Major Depressive Disorder might not differ much. For example the same sentence that describes ADHD in the introduction section of the study as “I have concentration difficulties because of my ADHD” is as tautological as saying “I have concentration difficulties because of my concentration difficulties,” could be applied to depression as "I have depression because of my Major Depression" as saying, "I have depression because of my depression." I only highlight this point as such statements may divert readers away from reviewing the study and understanding what it shows. | After some consideration, we decided to remove this part about ‘tautological reasoning’. We would like to briefly comment on this point, since I think diagnoses such as major depression are treated as a ‘condition’ in a more open and pluralistic fashion, both in the scientific literature, among laymen, and in clinical practise: For example, in cognitive psychotherapy the engine behind depression is conceptualized as maladaptive thoughts, in behavioral therapy as a lack of positive reinforcements from the environment, in biological psychiatry as a neurotransmitter impairment, in psychodynamic therapy due to maladaptive processing of unconscious emotions, and so on.  The Swedish National Council on Medical Ethics thus wrote in 2015 that it was problematic that diagnoses such as ADHD are (in Sweden) labeled ‘neuropsykiatriska’ [neuropsychiatric], since it can be perceived that the biological mechanisms have a greater explanatory value in ADHD than in other psychiatric diagnoses. It is our opinion that all psychiatric disorders have both biological, psychological, and sociological dimensions, and that the framing of some disorders as ‘neuropsychiatric’ makes them more conceptualized as biological entities and less as behavioral conditions, in comparison to other psychiatric diagnoses. We decided not to go to deep into this discussion, and therefor decided to remove this text, since as you highlighted it may divert readers away from understanding the study. |
| **Reviewer #3:** |  |
| Thank you for the opportunity to read and review this study. The study presents a new approach called IEN to better understand the distribution of ADHD. The approach involves a detailed analysis that goes beyond the national prevalence rate and reveals a significant variation in ADHD prevalence across demographic and socioeconomic categories. By adopting an intersectional perspective, the study shows that ADHD prevalence is influenced by various factors such as age, gender, immigration status, and socioeconomic standing. The study also emphasizes the importance of cautious interpretation of strata information to avoid perpetuating stigmas. |  |
|  |  |
| Despite the valuable insights gained from the stratified analysis, the study acknowledges the potential for stigmatization due to the detailed information. | Yes, we thought it was important to mention this. The risk of stigmatization is inherent in all epidemiological studies. As we have discussed elsewhere the risk of stigmatization is caused by attributing the same average group value to all individuals in the group, without considering the individual heterogeneity around the average. This is a problem in most epidemiological studies and originates an ethical dilemma as the aim of the epidemiological studies is to promote individual health. In many cases the benefits of epidemiological analyses outweigh the risk of stigmatization. However, a better way of handling this issue is by using measures of discriminatory accuracy (DA) as we do in our study. Measures of discriminatory accuracy informs you if the risk under investigation is universal (low DA) of clustered in specific groups (high DA). We have shortly discussed this issue in the discussion part of the revised manuscript, and we refer elsewhere for a more extend discussion.   - Merlo J, Mulinari S. Measures of discriminatory accuracy and categorizations in public health: a response to Allan Krasnik's editorial. Eur J Public Health. 2015;25(6):910. - Merlo J, Mulinari S, Wemrell M, Subramanian S V, Hedblad B. The tyranny of the averages and the indiscriminate use of risk factors in Public Health: The case of coronary heart disease. SSM - Population Health. 2017;3:684-98. |
|  |  |
| In addition, the study challenges previous research on the link between family income and ADHD and requires further exploration of potential explanations. Overall, the study's novel methodology, stratified analysis, intersectional perspective, and responsible data interpretation are commendable. However, addressing contextual interpretation, stigmatization concerns, cultural factors, practical applications of theory, and contradictions with existing research would further enhance the study's insights into the complex ADHD distribution landscape. (This was a suggestion for future work.) | Thank you for this suggestion. We added a note that future research can benefit from addressing contextual interpretation of symptoms and diagnosis, contradictions between different paradigms, and practical applications of the above insights. |
| **Reviewer #4:** |  |
| The paper presents a comprehensive analysis of ADHD prevalence in Sweden across different demographic and socioeconomic groups. Through a large-scale examination of over six million individuals, the authors unveil significant variations in ADHD risk based on age, gender, income, and country of birth. The paper had two primary aims: understanding the demographic and socioeconomic distribution of ADHD risk in Sweden, and contributing to the ongoing discourse on medicalization, particularly in relation to behavioral and social issues. I have the following comments/questions: |  |
| • The results provide valuable epidemiological information about the individuals diagnosed with ADHD. The authors do a great job of achieving aim 1 |  |
| • Although I agree with sentiment expressed by the authors about considering sociodemographic factors and their effects of ADHD diagnosis, their data does not do them any favors from an explanatory standpoint. The wide paradoxical outcomes reported across different strata do not provide a coherent explanation of how these sociodemographic factors affect the diagnosis. However instead of acknowledging this complexity and accepting that based on only there results they cannot conclusively confirm or refute the claim that increasing prevalence of ADHD can be explained away by sociodemographic factors, it appears that they double down and try and prove a point their data does not support. Thus the discussion section would benefit from such an acknowledgment | We understand the concern of the reviewer. However, traditional studies of socioeconomic differences based on simple dimensions like income or education or even a few ethnical or country of birth categorizations may hide considerable heterogeneity at the intersection of different dimension. The AIHDA approach we use, however, is able to reveal “unexpected” patterns of risk, which is an advantage of the analysis.  Taking your concern into account, we have in the revised manuscript been clearer with the fact that our results can not conclusively confirm or refute the claim that increasing prevalence of ADHD can be explained away by sociodemographic factors.  For example: “*This is not to say that the different strata in this study provide a more coherent explanation on* why *individuals become diagnosed. Rather, it is important to acknowledge the complexity of diagnoses entirely based on behavioral problems, and to highlight that some psychiatric categories should be approached with care, as blunt clinical tools*.” |
| **Reviewer #5:** |  |
| This is an interesting study that compared the risk of suffering from ADHD across 96 strata in the Swedish population. |  |
|  |  |
| Strengths of the study: |  |
| -The methodology of the study is strong. Being a population level study that has access to a large database (that covers 99% of inpatient and 80% of outpatient diagnoses) makes the specific results extremely well generalizable for a western country. |  |
| -Statistical analysis was also well described and is a valuable addition to the literature. |  |
|  |  |
| A few concerns: |  |
| -The introduction section would benefit from a rewrite and significant editing. There were multiple long run-on sentences. The same concepts were explained multiple times in different subsections of the introduction (like the discussion about theories from Ian Hacking, these are explained at length in both the 'ecological niches as a conceptual framework' and 'intersectional analysis' sub-sections). All of this could be condensed into one introduction section. | We have now made the introduction more concise by removing some sentences and by moving others from the introduction to the discussion part. Due to your concern, we have especially removed some of the introductory text on the theories of Ian Hacking, that were repetitive. |
| A couple of examples of sentences that need editing: |  |
| 1. page 2, first paragraph of introduction: "Without questioning the intentions of the proposal- people in these vulnerable areas do have a reduced tendency to seek this type of care - there is an all the more important observation to make." Could be broken down into easier and understandable language. | This was one of the sentences that we shortened. |
| 2. page 5, second paragraph: "Something that started out as a label for hyperactive children, ......" | This was one of the paragraphs where some wordings were transferred to the discussion. |
| -Alternatively, I would consider moving a lot of the introduction section into the discussion. Much of the concepts explained in the introduction belong much better in the discussion rather than setting up the study. This also fits in with the stated aims of the authors which include 1. the actual study looking at the distribution of ADHD and 2. a critical discussion on diagnosis and medicalization. | See above. |
| -In the discussion section, the authors highlight the differences between strata and attempt to explain these differences well. The roles of social context and understanding the ontology of ADHD are explained well and contribute to the discussion around this issue. |  |
| -However, the authors fail to offer a counter perspective to their arguments. As the authors point out in the introduction section, there have been investigations into function, structural and neurotransmitter alterations in the brains of those with ADHD. While the data may not be comprehensive and overwhelming indicative of one cause, there is enough underlying biological predisposition that cannot be dismissed entirely. While the social content that the authors describe can be potentially responsible for the increase in ADHD prevalence (the so called "epidemic"), this does not dismiss ADHD as an actual neurobiological condition. Rather it just helps us look at the parameters for diagnosis more critically. The clam the authors make around there being no 'natural' prevalence of ADHD that can be found is concerning. While our current tools/parameters for diagnosis are not ideal, this just seems to indicate that decades of scientific research into ADHD is pointless. This perspective is contradicted by clinical wisdom and also by the effectiveness of stimulant medication in treating ADHD symptoms (in the absence of other comorbidities). The authors do present a valuable perspective. However, this should be tempered and cognizant of other literature. The discussion section should be better balanced and appears rather skewed towards one side of this issue. | In the revised manuscript, we have strived to be a bit less polemic, removing or changing some wordings, and adding some more balance to the nature/nurture aspect of the discussion. After your recommendations, we have also mentioned and acknowledged the importance of biological predisposition. |
|  |  |
| Overall, a good study, could be a much better manuscript with some work. |  |
| **Reviewer #6:** |  |
| Please see attached world document for manuscript review edits and comments. There are no ethical concerns for the article. Authors conclude that ADHD's association to sociological component. I congratulate authors for same. |  |
| **Reviewer #7:** |  |
| Your paper presents some intriguing ideas, but the explanation of the intersectional ecological niche framework requires further clarification. Additionally, the summary could benefit from some concise editing. While discussing your work, individuals acknowledged its strengths, yet they expressed uncertainty regarding its limitations. Providing a more detailed explanation of these limitations is crucial. | We have done some editing to make the paper more concise throughout the script. We have tried to clarify the intersectional ecological niche framework as you requested, yet at the same time making this description less repetitive, as some reviewers commented on. We also discuss some limitations, mainly regarding the limited information that we had access to through the registers, and how we were not able to choose our variables of interest a priori. |
| ________________________________________ |  |
| 6. PLOS authors have the option to publish the peer review history of their article (what does this mean?). If published, this will include your full peer review and any attached files. |  |
| [NOTE: If reviewer comments were submitted as an attachment file, they will be attached to this email and accessible via the submission site. Please log into your account, locate the manuscript record, and check for the action link "View Attachments". If this link does not appear, there are no attachment files.] | We found no attachments to address. |
|  |  |
| While revising your submission, please upload your figure files to the Preflight Analysis and Conversion Engine (PACE) digital diagnostic tool, https://pacev2.apexcovantage.com/. PACE helps ensure that figures meet PLOS requirements. To use PACE, you must first register as a user. Registration is free. Then, login and navigate to the UPLOAD tab, where you will find detailed instructions on how to use the tool. If you encounter any issues or have any questions when using PACE, please email PLOS at figures@plos.org. Please note that Supporting Information files do not need this step. | Figures have now been uploaded to PACE and converted accordingly. |
