# Supplementary material for: Socioeconomic disparities in attention deficit hyperactivity disorder (ADHD) in Sweden: An intersectional ecological niches analysis of individual heterogeneity and discriminatory accuracy (IEN-AIHDA)
Source: PLoS One. 2023 Nov 20;18(11):e0294741. doi: 10.1371/journal.pone.0294741 (PMC10659213; doi:10.1371/journal.pone.0294741)
Supplement: Attachment — Submitted filename: ADHD REVIEW.docx [file pone.0294741.s006.docx]

1. I would like to thank you for the opportunity to review this article. The authors have done a wonderful job gathering the data and the sample size is excellent. There are clear graphs that are self explanatory.
2. The articles shows the difference in prevalence rates in different gender, age, country of birth and income groups in Sweden. The low income group in immigrant population has the lowest prevalence whereas middle and high income group immigrant (boys) have much higher prevalence. The native Swedes Low income group have higher prevalence compared to high income group natives. The high and medium income immigrant group showed a totally different trend compared to low income immigrant group which cannot be explained. There is definitely a role of social disadvantage as stress and trauma definitely contributes to increase in prevalence of ADHD diagnosis. But that does not explain why the trend reversed. The author has tried to explain it by saying that it probably depends on people’s attitudes and access to psychiatric treatment. However the article does not explain why the trend reverses with increase of income in immigrant population. Perhaps it will be good idea to understand where the immigrant population is coming from. Refugees from war afflicted countries with totally different cultures will have chronic stress, trauma and difficulty acculturing to the new country. Also children usually express frustration ( due to difficulties with new language and culture) by showing agitation which can be confused as impulsivity and aggression. Perhaps getting information on where the immigrants came from, educational level, perception of mental health in immigrants, cultural differences from countries of origin, trauma and substance use in population will be a valuable information to obtain
3. The article explains beautifully that incidence has increased but fails to address why is it so. The current research also takes inflammation into account for explaining increased prevalence. Perhaps the modern diet and modern day stresses in the childhood and prenatal period has something to do with it.

References

a. Pelsser LM, Buitelaar JK, Savelkoul HF. ADHD as a (non) allergic hypersensitivity disorder: a hypothesis. Pediatr Allergy Immunol. 2009 Mar;20(2):107-12. doi: 10.1111/j.1399-3038.2008.00749.x. Epub 2008 Apr 24. PMID: 18444966.

b. Anand D, Colpo GD, Zeni G, Zeni CP and Teixeira AL (2017) Attention-Deficit/Hyperactivity Disorder And Inflammation: What Does Current Knowledge Tell Us? A Systematic Review. Front. Psychiatry 8:228. doi: 10.3389/fpsyt.2017.00228

c. Chang JP, etal Cortisol, inflammatory biomarkers and neurotrophins in children and adolescents with attention deficit hyperactivity disorder (ADHD) in Taiwan. Brain Behav Immun. 2020 Aug;88:105-113. doi: 10.1016/j.bbi.2020.05.017. Epub 2020 May 8. PMID: 32418647.

4. The current research also hints toward the role of genetics in ADHD. There are also neuroimaging studies describing the differences in brain structure of ADHD vs non ADHD children. So the author blaming it on medicalization of ADHD and considering it as behavioral and social problem is perhaps too simplistic of an explanation.

References

a. Yadav SK et al. Genetic variations influence brain changes in patients with attention-deficit hyperactivity disorder. Transl Psychiatry. 2021 Jun 5;11(1):349. doi: 10.1038/s41398-021-01473-w. PMID: 34091591; PMCID: PMC8179928.
b. Stevens HE, Scuderi S, Collica SC, Tomasi S, Horvath TL, Vaccarino FM. Neonatal loss of FGFR2 in astroglial cells affects locomotion, sociability, working memory, and glia-neuron interactions in mice. Transl Psychiatry. 2023 Mar 11;13(1):89. doi: 10.1038/s41398-023-02372-y. PMID: 36906620; PMCID: PMC10008554.
