# Supplementary material for: Socioeconomic disparities in attention deficit hyperactivity disorder (ADHD) in Sweden: An intersectional ecological niches analysis of individual heterogeneity and discriminatory accuracy (IEN-AIHDA)
Source: PLoS One. 2023 Nov 20;18(11):e0294741. doi: 10.1371/journal.pone.0294741 (PMC10659213; doi:10.1371/journal.pone.0294741)
Supplement: Attachment — Submitted filename: Response to Reviewers.docx [file pone.0294741.s007.docx]

| 'Response to Reviewers' (rebuttal letter)  PONE-D-23-22871R1 (deadline Dec 01 2023 11:59PM)  Socioeconomic disparities in attention deficit hyperactivity disorder (ADHD) in Sweden: An intersectional ecological niches analysis of individual heterogeneity and discriminatory accuracy (IEN-AIHDA)  Lakshit Jain, MD  Academic Editor  PLOS ONE  Dear Editor,  Thank you very much for this opportunity and for transmitting to us your comments and the comments of the referees. We have considered and responded to all the commentaries as explaining in detail below. | |
| --- | --- |
| **EDITOR & REFEREES** | **AUTHORS** |
| **6. Review Comments to the Author** |  |
| **Reviewer #1** |  |
| Author has revised the manuscript with considering each reviewer comments and that has made the article better if not the best. | Thank you very much. |
| **Reviewer #5** |  |
| The authors have sufficiently addressed my concerns and have introduced a more nuanced and balanced perspective. The manuscript is much stronger as a result of the changes. I especially enjoyed reading the new discussion section. One of key points introduced in the revision is that interventions focused on ADHD should not just be limited to individual characteristics and rather focus on social conditions along with it. This is the highlight for this study. Good job! | Thank you very much. |
| **Reviewer #7** |  |
| The authors made appropriate changes in this revision. The research presents very good insight and better understanding about the ADHD and the new methods. | Thank you very much. |
| **Reviewer #8** |  |
| 1. I would like to thank you for the opportunity to review this article. The authors have done a wonderful job gathering the data and the sample size is excellent. There are clear graphs that are self explanatory. | Thank you very much for valuable comments. We have addressed them and responded below. |
| 2. The articles shows the difference in prevalence rates in different gender, age, country of birth and income groups in Sweden. The low income group in immigrant population has the lowest prevalence whereas middle and high income group immigrant (boys) have much higher prevalence. The native Swedes Low income group have higher prevalence compared to high income group natives. The high and middle income immigrant group showed a totally different trend compared to low income immigrant group which cannot be explained. There is definitely a role of social disadvantage as stress and trauma definitely contribute to increase in prevalence of ADHD diagnosis. But that does not explain why the trend reversed. The author has tried to explain it by saying that it probably depends on people’s attitudes and access to psychiatric treatment. However the article does not explain why the trend reverses with increase of income in immigrant population. Perhaps it will be a good idea to understand where the immigrant population is coming from. Refugees from war afflicted countries with totally different cultures will have chronic stress, trauma and difficulty acculturing to the new country. Also children usually express frustration (due to difficulties with new language and culture) by showing agitation which can be confused as impulsivity and aggression. Perhaps getting information on where the immigrants came from, educational level, perception of mental health in immigrants, cultural differences from countries of origin, trauma and substance use in population will be a valuable information to obtain  SOLUTION: Perhaps the lack of above information can be included in the limitation of study | This is an interesting comment where you address an important topic.  Not least, *why* does the low-income group in the immigrant population has such a low prevalence? One could only hypothesize that this group is less integrated into the majority society, in comparison with immigrants in the middle- and high-income groups. Such a lack of integration could mean a lesser utilization of health care (In the end, the prevalence rates depend on persons help-seeking behaviours, and on them becoming subjects to psychiatric assessments)  But also, as you mention, we have no data on what context these immigrants are coming from. Our data set only distinguishes between “born in Sweden” and “immigrant” unfortunately. From this perspective, we simply cannot analyse how coming from a war afflicted country, education level, cultural differences, and so on, relates to our data.  As you advised, we have now mentioned this as a limitation of the study. |
| 3. The article explains beautifully that incidence has increased but fails to address why is it so. The current research also takes inflammation into account for explaining increased prevalence. Perhaps the modern diet and modern day stresses in the childhood and prenatal period has something to do with it  SOLUTION: Perhaps the authors can also mention these findings in research paper. I am attaching a few reference articles for author.  References a. Pelsser LM, Buitelaar JK, Savelkoul HF. ADHD as a (non) allergic hypersensitivity disorder: a hypothesis. Pediatr Allergy Immunol. 2009 Mar;20(2):107-12. doi: 10.1111/j.1399-3038.2008.00749.x. Epub 2008 Apr 24. PMID: 18444966. b. Anand D, Colpo GD, Zeni G, Zeni CP and Teixeira AL (2017) Attention-Deficit/Hyperactivity Disorder And Inflammation: What Does Current Knowledge Tell Us? A Systematic Review. Front. Psychiatry 8:228. doi: 10.3389/fpsyt.2017.00228 c. Chang JP, etal Cortisol, inflammatory biomarkers and neurotrophins in children and adolescents with attention deficit hyperactivity disorder (ADHD) in Taiwan. Brain Behav Immun. 2020 Aug;88:105-113. doi: 10.1016/j.bbi.2020.05.017. Epub 2020 May 8. PMID: 32418647. | Thank you. While our aim is not primarily to study *why* the incidence has increased, but rather to present a detailed analysis on how ADHD as a category is linked to socioeconomic disparities, we agree with your recommendations and have now mentioned the findings on inflammation and modern diet/stresses:  “There is an abundance of research that has investigated correlates between subjects diagnosed with ADHD and potential biological abnormalities. Studies have reported an association with inflammatory processes [82], hypothesized a connection to foods and inhalants [83], or highlighted ADHD as part of a broader biological dysfunction in stress-relevant mechanisms [84].” |
| 4. The current research also hints toward the role of genetics in ADHD. There are also neuroimaging studies describing the differences in brain structure of ADHD vs non ADHD children. So the author blaming it on medicalization of ADHD and considering it as behavioral and social problem is perhaps too simplistic and narrow of an explanation. ADHD also has neurobiological underpinnings to it.  References a. Yadav SK et al. Genetic variations influence brain changes in patients with attention-deficit hyperactivity disorder. Transl Psychiatry. 2021 Jun 5;11(1):349. doi: 10.1038/s41398-021-01473-w. PMID: 34091591; PMCID: PMC8179928. b. Stevens HE, Scuderi S, Collica SC, Tomasi S, Horvath TL, Vaccarino FM. Neonatal loss of FGFR2 in astroglial cells affects locomotion, sociability, working memory, and glia-neuron interactions in mice. Transl Psychiatry. 2023 Mar 11;13(1):89. doi: 10.1038/s41398-023-02372-y. PMID: 36906620; PMCID: PMC10008554. | Thank you for this comment. Yes, there is probably a tangible role of genetics in ADHD. We have included the reference to Yadav et al, in a passage about how ADHD can be conceptualized as ‘neural abnormalities’.  What we want to emphasize in the article, however, is that genetically inherited traits should be separated from the questions: (1) what causes a specific person to become ‘functionally impaired’ in his/her individual context? (2) how is the line between normal and ‘abnormal’ conceptualized in a social, historic, and cultural context?  I think the following passages most adequately illustrates the stance of the authors:  “it is vital to distinguish between people's behavioral characteristics on the one hand, and why something becomes a disability, on the other. Even if a trait such as activity level might have a substantial genetic basis, this is not the same thing as having a disease [80]. The genetic findings from twin studies may simply display that children at risk of receiving an ADHD diagnosis more often have certain temperament factors.”  “People who have such traits to a lesser extent (but who also have childhood trauma, financial stress, low status in society, low self-esteem) arguably have a higher risk of fulfilling criteria D – problems with academic, social, and/or occupational functioning – and therefore being diagnosed with ADHD. In the end, a diagnosis is not a result of how much ‘ADHD traits’ a person has, but how well his or her life is working out. This functioning over time, i.e., adaptation to life, is apparently crucially intertwined with social variables. Such an awareness does not dismiss the fact that ADHD, as well as every other psychiatric category, contains a certain biological predisposition.” |
| SOLUTION: Please add it to conclusion section. There is a need for further understanding the genetic and neurological factors in addition to social correlates | While we acknowledge this need as one of several perspectives to be considered, we are not confident about it as a proposed conclusion of our discussion. In the literature on ADHD, there is already an overwhelming focus on genetic and neurological factors. One of the authors (Hornborg) has previously published a review article on this topic:  “A pubmed search performed on March 3rd, 2022, on ADHD and the relationship to genetics, genes or hereditary or inherited, resulted in a total of 46.994 articles published between 1990 and 2022. Research on adverse psychosocial circumstances and the presence of high stress in families where these children grow up is sparse in comparison... [there is a] biased knowledge production [that] also manifests in clinical practice where, as Singh (14) noted already 20 years ago, methylphenidate is often being used without  supporting interventions.”  Erlandsson, S., Hornborg, C., Sorbring, E., & Dauman, N. (2022). Is ADHD a way of conceptualizing long-term emotional stress and social disadvantage? *Frontiers in Public Health*, *10*, 1-14.  Our argument is that a continuing hunt for genetic and neurological correlates to a highly heterogenous and ambiguous category such as ADHD, does not seem to deliver much progress when it comes to public health or clinical practise, but rather risks concealing other more fruitful ways of approaching ADHD. For example, there is a need for more preventive measures on a societal level, but also for a shift from a nomothetic to a more idiographic perspective on the holistic life situation of these individuals. |
